# Fetal Cortical Abnormalities Identified on Ultrasound

**DOI:** 10.3390/diagnostics14212371

**Published:** 2024-10-24

**Authors:** Mara Rosner, Casey Reed, Aylin Tekes, Lindsey N. Goodman, Angie C. Jelin, Jena L. Miller, Michelle L. Kush, Ahmet A. Baschat, Lisa R. Sun, Jessica M. DeMay, Kristin Baranano

**Affiliations:** 1 Department of Gynecology and Obstetrics, Center for Fetal Therapy, Johns Hopkins University School of Medicine, Baltimore, MD 21287, USA; creed42@jhmi.edu (C.R.); lfromme1@jhmi.edu (L.N.G.); jmill260@jhmi.edu (J.L.M.); mkush1@jhmi.edu (M.L.K.); abascha1@jhmi.edu (A.A.B.); 2 Russell H. Morgan Department of Radiology and Radiological Science, Division of Pediatric Radiology, Johns Hopkins University School of Medicine, Baltimore, MD 21287, USA; atekes1@jhmi.edu; 3 Department of Gynecology and Obstetrics, Division of Maternal Fetal Medicine, Johns Hopkins University School of Medicine, Baltimore, MD 21287, USA; ajelin1@jhmi.edu; 4 Department of Neurology, Division of Pediatric Neurology, Johns Hopkins University School of Medicine, Baltimore, MD 21287, USA; lsun20@jhmi.edu (L.R.S.); kwb@jhmi.edu (K.B.); 5 Independent Researcher, Richmond, VA 23233, USA; jdemay@vwcenter.com

**Keywords:** cortical dysplasia, neuronal migration, ciliopathy, neurosonogram

## Abstract

Normal neuronal cell differentiation and migration is critical to brain formation, is rapidly occurring as the fetal brain develops, and peaks at the time of the routine ultrasound anatomic survey. Abnormalities in cortical migration can signify an underlying genetic abnormality or other fetal injury that can have a profound impact on future development. Although cortical migration peaks at 20–22 weeks, cortical migration abnormalities are rarely diagnosed at the time of the anatomic survey. We describe three cases of fetal cortical abnormalities in which prenatal ultrasound imaging was instrumental to making a prompt and accurate diagnosis in the mid-trimester and for guiding clinical counseling.

**Figure 1:**
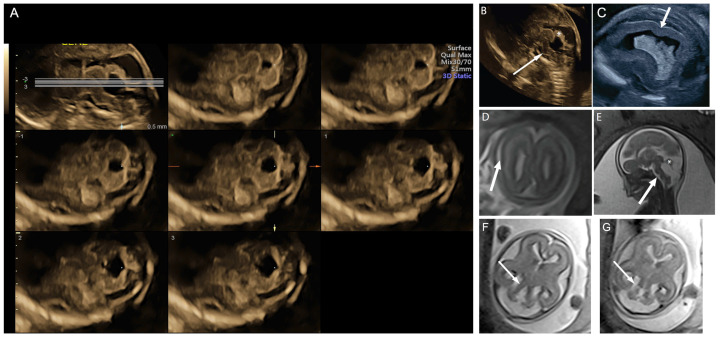
A 21-year-old G1P0 was referred at 16 weeks gestation for possible Blake’s pouch cyst. Her 12 week scan was notable for increased nuchal translucency. Neurosonogram at 16 weeks and 6 days tomographic ultrasound imaging (TUI) demonstrated splayed cerebellar hemispheres across multiple slice planes (lines = slice planes, upper left image) (**A**) and an abnormally rotated and dysplastic midline cerebellar structure, possibly representing the early fetal vermis (asterisk, (**B**)). These findings were readily differentiated from the referring diagnosis of Blake’s pouch cyst when additional ultrasound findings of hypersuclated parietal cortex, consistent with polymicrogyria (arrow, (**C**)) and hypoplastic brainstem (arrow, (**B**)), were also identified. Microarray was normal. MRI performed at 20 weeks and 4 days confirmed the ultrasound findings of polymicrogyria (arrow, (**D**)), abnormal brain stem (arrow, (**E**)), and hypoplastic and abnormally rotated midline cerebellar structure (asterisk, (**E**)). Thick and abnormal-appearing cerebellar peduncles were also noted (arrows, (**F**,**G**)). The patient elected termination. Trio exome sequencing revealed a compound heterozygous variant in *PIBF1* in the fetal specimen, which is consistent with a diagnosis of Joubert syndrome 33.

**Figure 2:**
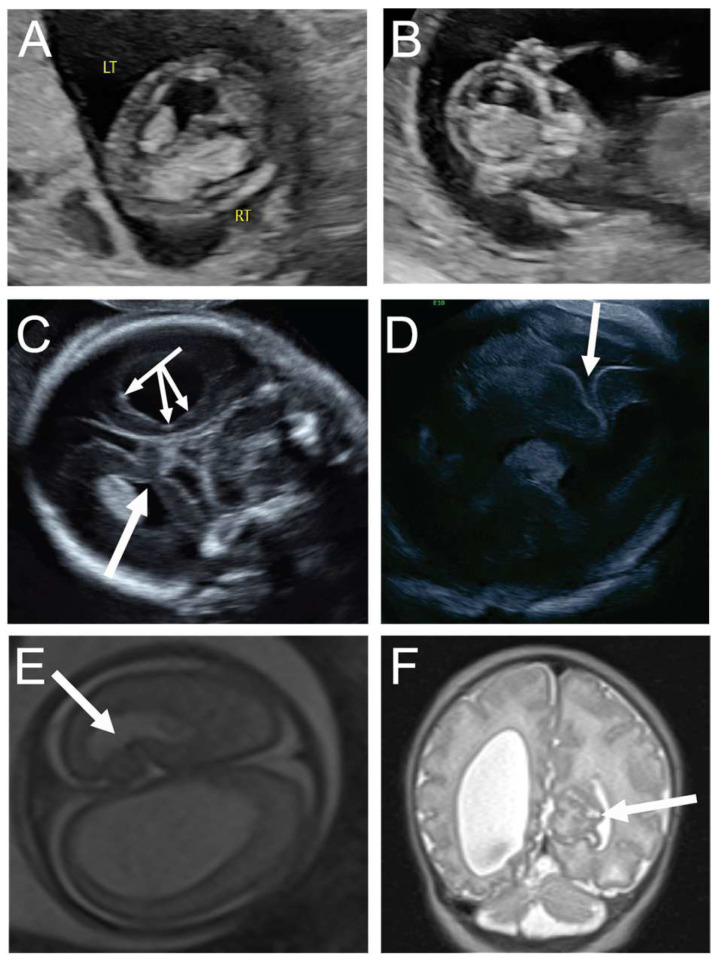
A 31-year-old G3P1 at 13 weeks and 1 day’s gestation was seen for first-trimester screening, and the fetus was noted to have an irregular, asymmetrically enlarged and mottled right choroid plexus, concerning for possible early right intraventricular hemorrhage and ventriculomegaly, note Rt = right side of fetus and Lt = left side of fetus (**A**,**B**). Maternal serum infectious and neonatal alloimmune thrombocytopenia workups were negative. Neurosonogram at 20 weeks and 0 days demonstrated the same severe unilateral right ventriculomegaly with stippling along the lining of the right ventricle, consistent with germinal matrix hemorrhage versus heterotopia (triple headed arrow, (**C**)), with additional findings of right periventricular white matter loss and a contralateral focal area of abnormal folding of the left mesial cortex, concerning for cortical dysplasia or polymicrogyria (single arrow, (**C**)). Fetal MRI confirmed right unilateral ventriculomegaly with evidence of right germinal matrix and intraventricular hemorrhage, as well as cortical abnormality in the left mesial occipital lobe (arrow, (**E**)). The mother opted to continue the pregnancy and declined amniocentesis. Ultrasound at 26 weeks and 5 days’ gestation showed progressive right ventriculomegaly with midline shift and continued prominence of contralateral cortical dysplasia, with possible progression to schizencephaly (arrow, (**D**)). She was delivered via cesarean at 36 weeks in the setting of preterm labor. Postnatal MRI demonstrated worsening of the cortical maldevelopment in the left mesial occipital lobe (arrow, (**F**)), a few scattered foci of periventricular nodular heterotropia, and abnormal vertical and shallow appearance of the right sylvian fissure. At two years of age, the child was meeting developmental milestones.

**Figure 3:**
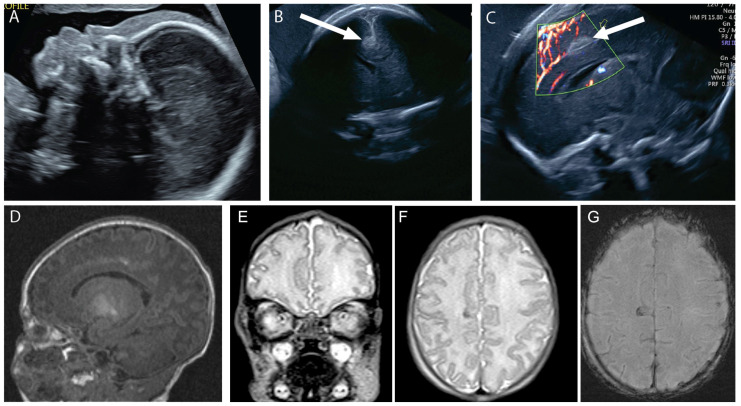
A 20-year-old G1P0 was referred for fetal midface hypoplasia (**A**). Neurosonogram at 25 weeks revealed a focal cortical abnormality in the right mesial cortex superior to the corpus callosum and just to the right of the falx (arrows, (**B**,**C**)). The patient’s cfDNA results were normal, and the mother declined amniocentesis. A fetal MRI at 26 weeks 2 days’ gestation revealed similar findings of mid-face hypoplasia and possible right medial cortical maldevelopment. Repeat MRI at 32 weeks’ gestation revealed similar findings (**D**). The baby was delivered vaginally at 37 weeks 2 days’ gestation and was admitted to the NICU. Postnatal MRI showed focal cortical abnormality in the mesial aspect of the right frontal lobe (**E**–**G**). The progression of the echogenic finding in the mesial cortical region is compatible with thrombosis of a superficial mesial cerebral vein, which is frequently associated with necrosis and gliosis of the adjacent cortical area, which can be seen in the T2 signal drop and the associated susceptibility artifact on susceptibility weighted imaging. The child’s SNP array was normal male. At one year of age, the child was undergoing physical and occupational therapy for a missed developmental milestone. By two years of age, the child was meeting milestones but had significant behavioral problems.

Advances in imaging technologies have allowed for earlier and improved diagnoses of cortical abnormalities in developing fetuses [1,2]. Fetal MRI after 20–22 weeks has been considered the gold standard for the identification of migration abnormalities, with higher sensitivity compared to ultrasound [3]. However, MRI cannot serve as the primary screening tool for cortical abnormalities due to the relatively advanced gestational age requirements and resource limitations [4,5]. Ultrasound is widely utilized as the first line for evaluating fetal anatomy and allows for serial evaluation. Image quality is also improving significantly, with attention being paid to advanced neurosonographic techniques [6,7]. Abnormalities of neuronal migration have been increasingly recognized as diagnosable by sonography, even prior to 20 weeks.

Cortical maldevelopment has many varied appearances on ultrasound. However, characteristic features can be observed if careful attention to the cerebral cortex is applied [1,5,6]. In all our cases, cortical abnormalities were initially suspected on neurosonogram and subsequently confirmed on MRI. Initial suspicion of brain or facial abnormality noted at the time of screening ultrasound facilitated care for each patient.

In the first case, the most prominent finding was a cystic dilation of the posterior fossa at 15 weeks, which was thought to be a possible Blake’s pouch cyst, a potentially favorable prognosis. However, the hypersulcated cortex at 16 weeks was an early clue that the fetus had a more severe and global disorder of neuronal migration. The differential diagnosis of global disorders of cortical maldevelopment, impacting both the cortex and posterior fossa, include conditions along the alfa-dystroglycanopathies, other Jouberts syndrome-related disorders, or tubulinopathy spectra. These disorders have characteristic neuroanatomic findings that may allow them to be differentiated during fetal life, particularly with the help of fetal MRI, but ultimately, genetic and/or postnatal clinical diagnosis can be required for confirmation [8].

In the second case, intraventricular hemorrhage was suspected on first-trimester ultrasound. Monitoring progression throughout the trimesters was possible on ultrasound, and it revealed not only significant ipsilateral cortical volume loss with severe unilateral ventriculomegaly but also the unanticipated finding of contralateral cortical dysplasia, consistent with cortical dysplasia or polymicrogyria, without evidence of volume loss. These findings support the theory that perinatal ischemia from vascular disruption can lead to schizencephaly and cortical dysplasia [9] and may impact contralateral cerebral cortex. Given that the findings suggest perinatal ischemia/vascular disruption, potentially causative genetic etiologies such as COL4A1, COL4A2, JAM3, and TREX1, should be considered and are best evaluated with whole-exome sequencing.

The third case demonstrates a focal lesion in an otherwise normal-appearing brain in the setting of an abnormal facial profile, consistent with focal cortical abnormality. The findings on the neurosonogram were the indication for a fetal MRI that confirmed the presence of a focal area of abnormal cortex and led to the initiation of developmental follow up for this child.

In summary, familiarity with the appearance of neuronal migration abnormalities on ultrasound can assist in the early diagnosis of global disorders of cortical development, can identify progression of hemorrhagic sequelae in the fetus, and can identify apparently isolated focal cortical abnormality. Neurosonographic follow up also allows for an improved understanding of serial changes that occur during fetal development of cortical malformations.

## Data Availability

The original contributions presented in the study are included in the article, further inquiries can be directed to the corresponding authors.
